# Indolaminergic System in Adult Rat Testes: Evidence for a Local Serotonin System

**DOI:** 10.3389/fnana.2020.570058

**Published:** 2021-02-19

**Authors:** Francisco Jiménez-Trejo, Isabel Coronado-Mares, Cristian Arriaga-Canon, Luis A. Herrera, Bladimir Roque-Ramírez, Margarita Chávez-Saldaña, Julio Rojas-Castañeda, Marco Cerbón, Rosa M. Vigueras-Villaseñor

**Affiliations:** ^1^Instituto Nacional de Pediatría, Ciudad de México, Mexico; ^2^Facultad de Química, Universidad Nacional Autónoma de México, Ciudad Universitaria, Ciudad de México, Mexico; ^3^Instituto Nacional de Cancerología, Ciudad de México, Mexico; ^4^Unidad de Investigación Biomédica en Cáncer, Instituto Nacional de Cancerología-Instituto de Investigaciones Biomédicas, Ciudad de México, Mexico; ^5^Instituto Nacional de Medicina Genómica, Ciudad de México, Mexico

**Keywords:** serotonin, 5-HT, indolamine, testes, serotoninomics, spermatogenesis

## Abstract

Serotonin (5-HT) is member of a family of indolamine molecules that participate in a wide variety of biological processes. Despite its important role in the regulation of local blood systems, little is known about the physiological function of 5-HT in reproductive organs, its functional implications, and its role in the reproduction of mammals. In the present work, we evaluated the localization and distribution of 5-HT (using histochemical analysis of indolamines) and different components of the serotoninergic system in rat testes. We detected local synthesis and degradation through immunofluorescence and western blot analyses against the TPH1, MAO_A_, 5-HT_T_, and VMAT1 serotonin transporters. We also identified the localization and distribution of the 5-HT_1B_, 5-HT_2A_, and 5-HT_3A_ receptors. RT-PCR results showed the presence of the Tph1, Maoa, Slc6a4, and Htr3a genes in testes and in the brain stem (Tph1 was used as a negative control). High-performance liquid chromatography was used to determine the presence of 5-HT and the activity of tryptophan hydroxylase in testes homogenates *in vitro*. Our observations suggest that TPH1 activity and local 5-HT synthesis befall in rat testes. We propose that 5-HT could participate in the regulation of testosterone synthesis and in the spermatogenesis process via local serotoninergic system. However, more studies are needed before concluding that rat testes, or those of other mammals, contain an active form of tryptophan hydroxylase and produce 5-HT.

## Introduction

Indolamines are a family of neurotransmitters that share a common molecular structure and are biologically synthesized from the essential amino acid tryptophan. They function as neurotransmitters and neuromodulators in the central nervous system (CNS), whereas in the periphery they serve as neurohormones such as catecholamine, serotonin, and melatonin (Rapport et al., 1948; Young, 2007).

Since its discovery in 1948 by Maurice Rapport, Arda Green, and Irvin Page, serotonin [5-hydroxytryptamine; C_10_H_12_N_2_O (5-HT)] has been shown to be involved in multiple functions in a broad range of living beings (Berger et al., 2009). Its influence has been observed in diverse mammalian systems, including the gastrointestinal, cardiovascular, pulmonary, and genitourinary systems, as well as the CNS (MacLean et al., 2000; Liu et al., 2002; Gaspar et al., 2003; De Ponti, 2004; Jiménez-Trejo et al., 2007, 2012, 2018). 5-HT modulates various physiological functions, including emotions, cognition, sleep, arousal, feeding, temperature regulation, and pain (Gaspar et al., 2003).

L-tryptophan (L-Trp) is an essential amino acid and precursor for the biosynthesis of 5-HT in the brain and in peripheral tissues. In the brain, L-Trp is hydroxylated in serotonergic neurons by the action of the enzyme tryptophan hydroxylase 2 (TPH2; EC 1.14.16.4, TrpOH). By contrast, TPH1 is expressed predominantly in peripheral tissues. These enzymes are encoded by two different genes and are the rate limiting enzymes for 5-HT biosynthesis in central and peripheral organs (Walther and Bader, 2003; Walther et al., 2003). 5-hydroxytryptophan is then decarboxylated to 5-HT (Gaspar et al., 2003).

In mammals, a few studies have reported the presence and distribution of 5-HT in reproductive organs from both male and female individuals, but its functional contributions remain obscure (Fujinoki, 2011; Jiménez-Trejo et al., 2012, 2013; Vela-Hinojosa et al., 2013). In a first attempt to decipher its role, we have described the distribution of several elements of the serotoninergic system in the testes during spermatogenesis, the mating period, fertilization, and the sperm maturation processes occurring in the caput epididymis (Jiménez-Trejo et al., 2007, 2012, 2018).

Previous studies have described the diverse effects of 5-HT in gonads, including regulation of testicular growth, cAMP production, and testosterone production (Tinajero et al., 1993a; Frungieri et al., 2002). Furthermore, 5-HT has been implicated in the modulation of androgen production in rats (Dufau et al., 1993), Syrian hamsters (Frungieri et al., 1999, 2002), and bats (Jiménez-Trejo et al., 2013). Interestingly, 5-HT contributes to the regulation of testosterone release from Leydig cells to the interstitial zone (Tinajero et al.,1993a,b). *In vitro* studies have documented the ability of pharmacological concentrations of 5-HT to modulate sperm motility and fertility rates in animals (Meizel and Turner, 1983; Parisi et al., 1984; Stephens and Prior, 1992; Fujinoki, 2011; Jiménez-Trejo et al., 2012).

In testes, it has been previously reported that 5-HT is mainly located in Leydig cells in the interstitial zone, in mast cells situated in the testicular capsule, in testicular blood flow, and in some nervous fibers (Campos et al., 1990; Tinajero et al.,1993a,b; Collin et al., 1996; Frungieri et al., 1999). Apparently, Leydig cells express TPH1. Although this has been demonstrated in cell culture (Tinajero et al.,1993a,b), the presence of local synthesis has not been confirmed (Tinajero et al., 1993a; Frungieri et al., 1999, 2002). In Leydig cell cultures, 5-HT was also shown to act synergistically with corticotropin-releasing factor to negatively regulate the synthesis of testosterone in an autocrine manner through 5-HT_2_ receptors (Tinajero et al., 1992).

However, the potential effects of 5-HT and its components during spermatogenesis have not yet been determined, although this indolamine may be involved in urogenital pathologies such as varicocele, erectile dysfunction, premature ejaculation, infertility, and low sperm count in humans and probably in other animal species (Gonzales et al., 1989; Gonzales et al., 1992; Nørr et al., 2016).

In the present work, we aimed to confirm the cellular distribution of 5-HT in rat testes. We also sought to detect serotonin-related proteins, including the TPH1 and monoamine oxidase A (MAO_A_) enzymes; 5-HT_T_ and VMAT1 transporters; and 5-HT_1B_, 5-HT_2A_, and 5-HT_3A_ receptors. Using histochemical, immunolocalization, and molecular techniques, we showed that 5-HT is present in rat testes, specifically, in vesicles located inside the cytoplasm of Leydig cells, but also in both cytoplasmic vesicles and prolongations (ramifications) that run toward the lumen in specific zones along seminiferous tubules, presumptively round spermatids. In addition, we found that Leydig cells were positive against TPH1, 5-HT_2A_ receptor, and 5-HT_T_ but not MAO_A_. 5-HT receptors, MAO_A_, and both the 5HT_T_ and VMAT1 transporters showed regionalized expression within seminiferous tubules. *In vitro*, we demonstrated TPH activity and the presence of 5-HT in testes homogenates using high-performance liquid chromatography (HPLC). These results further support the existence of a local serotoninergic system in rat testes, which could modulate both the steroidogenesis and spermatogenesis processes.

## Materials and Methods

Experiments were conducted in adult male Wistar rats (120 days old), bred and raised at the animal facilities of the Instituto Nacional de Pediatría, Mexico. The rats were kept under controlled temperature (22°C), humidity, and photo-period (light on at 7:00, light off at 18:00 GMT-6) conditions. Rats had free access to food and water and were sacrificed at 14:00 h. Animal handling and experimentation followed the Guidelines for Care and Use of Laboratory Animals published by the National Institutes of Health. Local Animal Rights Committees of the Institute approved the protocols: 014/2016 and 046/2019.

### Isolation of RNA and RT-PCR Assays

Total RNA extractions from rat testicles were performed using TRIzol reagent (Invitrogen, Carlsbad, CA, United States). The cDNA was obtained by reverse transcription from 2 mg of total RNA using a SuperScript^TM^ First-Strand Synthesis kit (Invitrogen), and was used for expression analysis of the *Tph1*, *Maoa*, *Htr3a*, *Slc6a4*, and *Gapdh* genes. PCR was performed using the following primers:

*Tph1* forward 5′-AGACACCTGCCACGAACTCT-3′ and reverse 5′-TGCTTGCACAGTCCAAACTC-3′;

*Maoa* forward 5′-AGCAAGACACGCTCAGGAAT-3′ and reverse 5′-CCACAGGGCAGATACCTCAT-3′;

*Htr3a* forward 5′-CATGTATGCCATCCTCAACG-3′ and reverse 5′-GGGATGGACAATTTGGTGAC-3′;

*Slc6a4* forward 5′-AAAGGCGTCAAAACATCTGG-3′ and reverse 5′-TCTACCCACACCCCTGTCTC-3′;

*Gapdh* forward 5′-AGCCACATCGCTCAGACAC-3′ and reverse 5′-GCCCAATACGACCAAATCC-3′.

*Gapdh* was used as an endogenous gene expression control. PCR conditions were as follows: (1) pre-incubation for 5 min at 95°C; (2) 33 cycles, each consisting of denaturation for 30 s at 95°C, annealing for 30 s at 63°C, and extension for 30 s at 72°C; and (3) a final extension period of 7 min at 72°C.

### Modified Falck-Hillarp Histochemistry

Testes of anesthetized animals (sodic pentobarbital, 45 mg/kg of body weight; Pfizer, New York, NY, United States) were dissected, and epididymis and fat were removed. Testes were dissected by incising the medial line of the scrotum. The testicular capsule was exposed, and the efferent ducts and spermatic cord were cut. The isolated testes were rapidly washed with 0.9% NaCl, excess water was removed, and the tissues were frozen in 2-methyl butane pre-chilled with dry ice and stored at –70°C until use. A longitudinal section (8 μm) was cut on a cryostat, mounted onto gelatin-coated slides, and fixed in paraformaldehyde (4%) dissolved in phosphate buffer (0.1 M PBS, pH 7.4).

After vacuum-sealing of the slides, tissues were placed in a solution containing 1% paraformaldehyde (PFA) and 8% glyoxylic acid (HO_2_CHCO_2_H) in phosphate buffer solution (0.1 M PBS, pH 7.4) at 4°C for 10 min. Control slides were placed in PBS only. Samples were dried and immediately placed at 100°C for 10 min. Some sections were counterstained with Hoechst stain for 10 min in order to visualize the cell nuclei. The sections were mounted with a special commercial medium for fluorescence (Fluorescence Mounting Medium, DAKO, DakoCytomation, Carpinteria, CA, United States). Slides were observed with an epifluorescence microscope using a fluorescein filter. Molecules were considered positive for 5-HT if a yellow fluorescent signal was detected (Hökfelt, 2010; Jiménez-Trejo et al., 2013).

### Immunodetection of Serotonin Markers

Tissues were mounted on gelatin-coated slides and fixed in 4% PFA dissolved in PBS for 30 min. For the immunohistochemistry, endogenous peroxidase activity was first quenched by incubating the slides in a 3% hydrogen peroxide solution. After washing, the sections were incubated with blocking solution (3% bovine serum albumin, 0.3% Triton X-100 and 0.025% sodium azide in PBS) for 4 h at room temperature. Then, the sections were incubated overnight at 4°C with the appropriate primary antibody. The following information about the specificity and cross-reactivity of each antibody was provided by the suppliers.

(1) Rat monoclonal anti-5-HT (1:100; YC5/45; GTX31100; GeneTex, Inc., Irvine, CA, United States); this antibody recognizes 5-HT and does not cross-react with 5-hydroxyindoleacetic acid, GABA, noradrenaline, 5-hydroxytryptophan, carnosine, or melatonin.
(2) Anti-MAO_A_ (1:100; H-70; sc-20156; Santa Cruz Biotechnology Inc., Santa Cruz, CA, United States) is a rabbit polyclonal antibody raised against amino acids 458–527 of human origin.
(3) Anti-TPH (1:50; C-20; sc-15116) is an affinity-purified goat polyclonal antibody raised against a peptide mapping within an internal region of human origin.
(4) Anti-5-HT_1B_ (1:100; SR-1B; M-19; sc-1461) is an affinity-purified goat polyclonal antibody raised against a peptide mapping at the C-terminus of mouse origin.
(5) Anti-5-HT_2A_ (1:100; SR-2A; A-4; sc-166775) is a mouse monoclonal antibody raised against amino acids 1–75 mapping within an N-terminal extracellular domain of human origin.
(6) Anti-5-HT_3A_ (1:50; SR-3A, C-20; sc-19152) is an affinity-purified goat polyclonal antibody with epitope mapping near the C-terminus of human origin.
(7) Anti-5-HT_T_ (1:50; ST (N-14; sc-14514) is an affinity-purified goat polyclonal antibody raised against a peptide mapping at the N-terminus of ST of human origin.
(8) Anti-VMAT1 (1:100; G-12; sc-166391) is a mouse monoclonal antibody raised against amino acids 44–143 mapping near the N-terminus of human origin.

After three washes with PBS, sections were incubated for 2 h at room temperature with the corresponding secondary antibodies coupled with peroxidase enzyme or fluorochromes (Santa Cruz Biotechnology Inc.), diluted to 1:500 in blocking solution. Washed slides were cover-slip-embedded with fluorescence mounting medium (DAKO). In control experiments, slides were incubated with pre-immune serum, or the primary antibodies were omitted. Experiments were performed in triplicate. The sections were visualized and images were acquired using a Nikon E600 fluorescence microscope equipped with a digital camera (Digital Sight DS-5M, Nikon, Melville, NY, United States). Images were digitized and figures were produced using Adobe Photoshop 10.0.1 or Adobe Photoshop CS5.1 version 12.1 X64 (Adobe Systems Incorporated, San Jose, CA, Unites States). Expression estimation was performed based on images captured from three different tests for each marker used. Although this is not an unbiased method, the cell expression density results are fully comparable with those obtained using the dissector method (Wilson et al., 1998). We analyzed testes from at least three animals. We did not perform statistical tests for this evaluation.

### Western Blot to Reconfirm Serotonin-Related Proteins

Tissue samples were homogenized in a buffer containing Trizma hydrochloride (Tris–HCl; 0.05 mol, pH 7.4), dithiothreitol (1 mmol), and acetic acid [ethylene bis(oxyethylenenitrilo)] tetraacetic acid/ethylene glycol-bis(2-aminoethyl ether)-N,N,N′,N′-tetraacetic acid (EGTA; 1 mmol), supplemented with a mixture of protease inhibitors (Complete, EDTA-free, Roche-Mannheim, Germany). Samples (85 μg of protein per well) diluted in Laemmli solution were electrophoresed under reducing conditions (5% β-mercaptoethanol) in sodium dodecyl sulfate polyacrylamide gels (12%) at 100–120 V for 4.5 h, following the protocol described previously (Jiménez-Trejo et al., 2007). We used pre-stained molecular weight markers to determine the relative mobility of proteins (Kaleidoscope; Bio-Rad, Hercules, CA, United States). Following electrophoresis, gels were equilibrated, and the proteins were transferred to nitrocellulose sheets (Bio-Rad) at 350 mA for 1 h at 4°C and incubated with the following polyclonal antibodies: anti-TPH1, anti-MAO_A_, anti-5HT_1B_, anti-5-HT_2A_, anti-5-HT_3A_, and anti-VMAT1 (1:1000) overnight at room temperature.

Membranes were incubated with IgG secondary antibodies conjugated with horseradish peroxidase (1:7000, Vector) for 1 h at room temperature. Finally, peroxidase activity was examined using a chemiluminescence-based detection kit according to the protocol suggested by the manufacturer [ECL, Amersham Pharmacia-Biotech, Buckinghamshire, United Kingdom or DAB + Chromogen (DAKO)]. Membranes were exposed to DAB for 2 min at room temperature. Images were captured and digitized using Photoshop SC6.

### HPLC to Evaluate TPH1 Activity and 5-HT Concentration in Male Testes

Testes samples were obtained from 120-days-old animals to evaluate local synthesis during sexual maturation. Tissue samples from the five animals described were dissected on an ice-chilled plate, weighed, frozen in liquid nitrogen, and stored at –68°C until use. The activity of TPH in the testes was estimated by measuring the production of 5-hydroxytryptophan, following a protocol described previously (Jiménez-Trejo et al., 2007). 5-hydroxytryptophan was then quantified using a fluorescence detector (Waters). The standard used was 5-hydroxytryptophan (100, 2.5, and 0.0625 ng/20 mL). Results for TPH1 activity are expressed in nanomoles of product per milligram of protein per hour (nmol/mg protein/h), and 5-HT concentrations are expressed as pg/mg of tissue.

## Results

**FIGURE 1:**
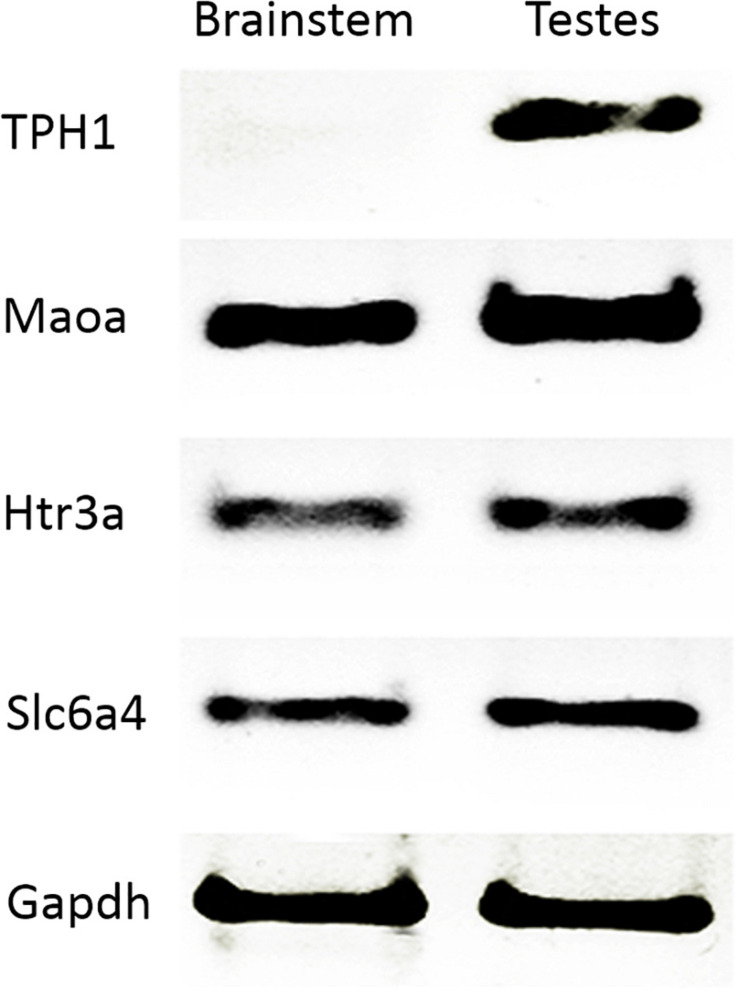
Transcripts of the serotonin system expressed in rat testes. RT-PCR confirmed gene expression related to 5-HT. We used brain stem as a positive control in testes (Maoa, Htr3a, and Slc6a4), and tph1 as a negative control. Gapdh was used as a constitutive gene control.

In order to confirm the presence of some serotoninergic system components in the testes, we analyzed gene expression through RT-PCR. For this, we used brain stem as the positive control. Concordantly, Tph1 (TPH1) was not expressed in the brain stem but in the testes. Maoa (MAO_A_), Slc6a4 (5-HT_T_), and Htr3a (5-HT_3A_) transcripts were present in both adult rat testes and brain stem (Figure 1). Gapdh mRNA was used as an expression control (Supplementary Figure 1 shows a complete gel where RT-PCR was standardized; Supplementary Figure 2 shows all RT-PCR amplicons and the molecular weight marker requested).

**FIGURE 2:**
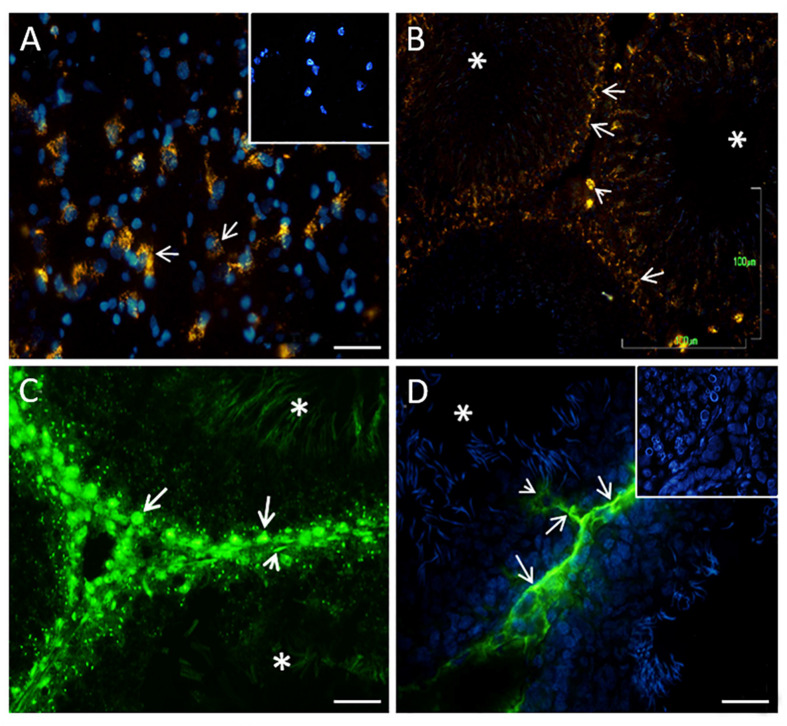
Digital photomicrographs showing histochemical analyses of indolamines in the brain stem [**(A)**, positive control] and rat testes **(B)**. In both tissues, structures containing 5-HT in the cellular cytoplasm are shown [arrows in part **(A)**]. The inset in **(A)** represents a negative control. The arrowhead in **(B)** shows Leydig cells located in the interstitial zone, apparently spermatogonia cells present a histochemical reaction (arrows). The asterisk represents the lumen of the seminiferous tubules. **(C)** 5-HT immunofluorescence in the tubules, with the appearance of spermatogonia cells (arrow), cell myoids (arrowhead), and sperm localized in the lumen (asterisks). **(D)** Positive reactivity in the interstitial zone and axon-like structures that penetrate the tubules zone. The inset in **(D)** shows the negative control. Scale bars **(A,C,D)**: 20 μm; **(B)**: 100 μm.

Using the histochemical technique to detect 5-HT, we observed vesicle-like structures filled with 5-HT in the cytoplasm of the brain stem neurons used as a positive control (arrows in Figure 2A). The inset in Figure 2A shows the negative control. A similar pattern of expression was found in the testes. A high-intensity signal was detected in Leydig cells located in the interstitial zone (arrowhead) and, interestingly, in disperse, positive vesicles along some seminiferous tubules in spermatogonia-like cells (arrows in Figure 2B) and some Sertoli cells with cytoplasmic prolongations. We corroborated this pattern of 5-HT distribution with immunofluorescence against 5-HT; Figure 2C shows the signal into the tubules, with the appearance of spermatogonia cells (arrows), preleptotene spermatocytes, and Sertoli and myoid cells (arrowhead). In the lumen, the sperm showed immunostaining (asterisks). Interestingly, in some tubules, we observed positive reactivity in the interstitial zone in axon-like or cytoplasmic prolongations of Leydig cells (arrows) that penetrated contacting cells near the tubular lumen (arrowhead in Figure 2D). The inset in Figure 2D corresponds to the negative control. Asterisks in Figures 2B–D correspond to tubular lumen (*n* = 3).

**FIGURE 3:**
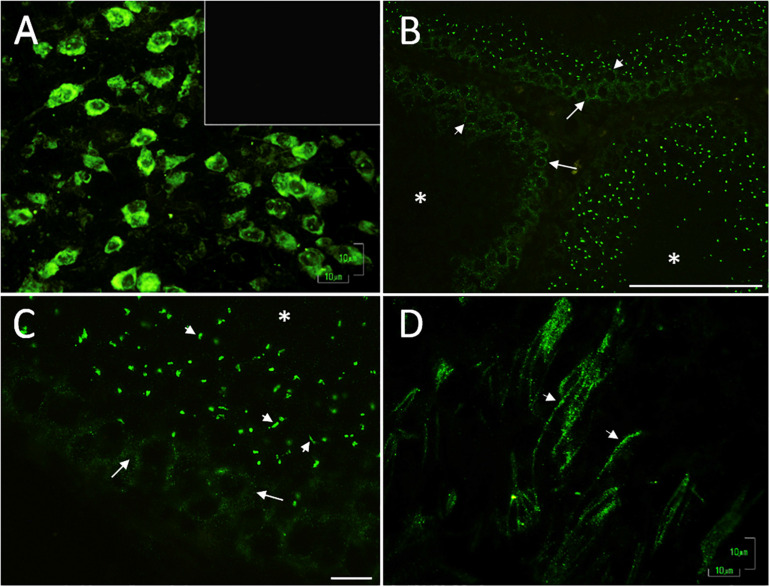
Digital photomicrographs illustrating the pattern of cell staining for MAO_A_, expressed in the cytoplasm of brain stem neurons **(A)** used as a positive control; inset corresponds to the negative control. In rat testes, we found vesicle-like signals in spermatogonia cells (arrows) and spermatocytes (arrowheads); located inside seminiferous tubules **(B)**. **(C)** Close-up view of the signal in spermatocytes (arrows) and cytoplasmic droplet-like structures (arrowheads). Asterisks indicate the lumen in **(B,C)**. **(D)** Positive reaction observed in sperm flagella localized in the lumen (arrowheads). The controls were testes slides incubated with pre-immune serum [inset in **(A)**]. Scale bar **(A,C,D)** 10 μm; **(B)** 100 μm.

Next, we tested for the presence of 5-HT-related proteins and determined their distribution in the testes. As expected, MAO_A_, the enzyme responsible for the catabolism of 5-HT, was found to be abundantly expressed in the cytoplasm of the neurons (arrows) belonging to the brain stem (positive control) (Figure 3A). In the panoramic view of slides of rat testes, we observed a vesicle-like positive signal in spermatogonials (arrows) and spermatocytes (arrowheads), located in the seminiferous tubules (Figure 3B). To increase the signal, we obtained a close-up view of the observed signal in spermatocytes (arrows); vesicles localized in the lumen were observed, with the appearance of residual cytoplasmic droplets (Figure 3C). Interestingly, the sperm localized in the lumen showed immune expression in the flagellum (arrowheads in Figure 3D). In control experiments, testes slides were incubated with pre-immune serum, and no positive immunoreactivity was detected (inset in Figure 3A). Asterisks indicate tubular lumen in Figures 3B,C.

**FIGURE 4:**
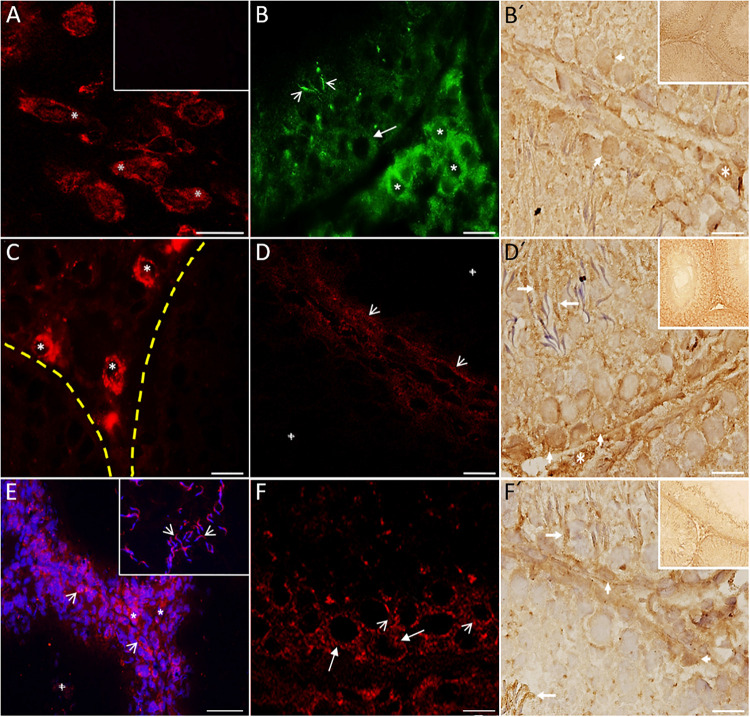
Digital photomicrographs showing 5-HT system markers present in rat testes. Brain stem was used as a positive control. **(A)** Signal in neuron cytoplasm (asterisks). The inset corresponds to the negative control. **(B)** Immunofluorescence reactivity for 5-HT_1B_ in Leydig cells (asterisks) and spermatogonia localized in the basal region of several seminiferous tubules (arrow). Sperm heads also show immunoreactivity (arrowheads). Immunohistochemistry results **(B′)** show the 5-HT_1B_ receptor in Leydig cells (asterisk) and some spermatogonia attached to the basement membrane (arrowheads). By contrast, TPH1 was found to be expressed in Leydig cells (asterisks) and also weakly in the basal membrane of seminiferous tubules **(C)**. The yellow dashed line indicates the area of the basal membrane of the seminiferous tubules. **(D)** Slight staining of 5-HT_2A_ in the basal membrane (arrowheads). Immunohistochemistry results **(D′)** show positive immunoreactivity against the 5-HT_2A_ receptor in Leydig cells (asterisk), some spermatogonia (arrowheads), and sperm (arrows). **(E)** 5-HT_T_ was located in both Sertoli (arrowheads) and Leydig cells (asterisks). The inset in part **(F)** shows immunoreactivity to 5-HT_T_ in the acrosomal region of sperm located in the lumen (arrowheads). The 5-HT_3A_ receptor was located inside the seminiferous tubules **(F)**, mainly in spermatogonia (arrows) and spermatocytes (arrowheads). Immunohistochemistry results **(F′)** shows a positive reaction against the 5-HT_3A_ receptor in some Sertoli (arrowheads) and sperm heads (arrows). The cross in **(D,E)** represents a tubular lumen. Insets in **(B′,D′,F′)** give a panoramic view of the tubular region. Scale bar = 10 μm.

Immunofluorescence studies were used to evaluate the presence and distribution of the 5-HT receptors (5-HT_1B_, 5-HT_2A_, and 5-HT_3A_) in testes. We used brain stem as a positive control for several markers related to the 5-HT system and to demonstrate the intense signal in neuron cytoplasm (i.e., 5-HT_1B_; asterisks in Figure 4A). No reaction was observed in the seminiferous tubules incubated with the conjugated antibody only (inset in Figure 4A). We observed an intense signal for 5-HT_1B_ (Figure 4B) in the basal region of several seminiferous tubules, mainly in spermatogonium (arrow) in the interstitial zone in Leydig cells (asterisks) and sperm heads (arrowheads). Figure 4B’ shows a photomicrograph of seminiferous tubules with positive immunohistochemical signals against the 5-HT_1B_ receptor in Leydig cells (asterisk), and some spermatogonial cells attached to the basement membrane (arrowheads). In contrast to TPH1, the rate-limiting enzyme in the peripheral 5-HT synthesis pathway was found to be highly expressed in Leydig cells (Figure 4C; asterisks); the yellow dashed line indicates the area of the basal membrane of the seminiferous tubules. As shown in Figure 4D, 5-HT_2A_ was found in the basal membrane and in a more intense elongated process, which ran along the basal membrane (arrowheads; crosses represent the tubular lumen). Figure 4D′ shows positive immunoreactivity against the 5-HT_2A_ receptor in Leydig cells (asterisk), whereas in the region of the basement membrane, some spermatogonia (arrowheads) and sperm showed an apparent signal (arrows). As shown in Figure 4E, 5-HT_T_ was localized in both Sertoli cells (arrowheads) and Leydig cells (asterisks). We observed 5-HT_T_ immunoreactivity in the acrosomal region of sperm located in the lumen of seminiferous tubules (arrowheads in the inset). The cross represents a tubular lumen. Figure 4F shows the 5-HT_3A_ receptor located in cells in the seminiferous tubule, mainly in spermatogonia (arrows) and preleptotene spermatocytes (arrowheads). The immunohistochemistry results in Figure 4F′ show a positive reaction against the 5-HT_3A_ receptor in some Sertoli cells (arrowheads), Leydig cells (asterisks), and sperm heads (arrows). Insets in Figures 4B′,D′,F′ show panoramic views of the tubular region (*n* = 3).

**FIGURE 5:**
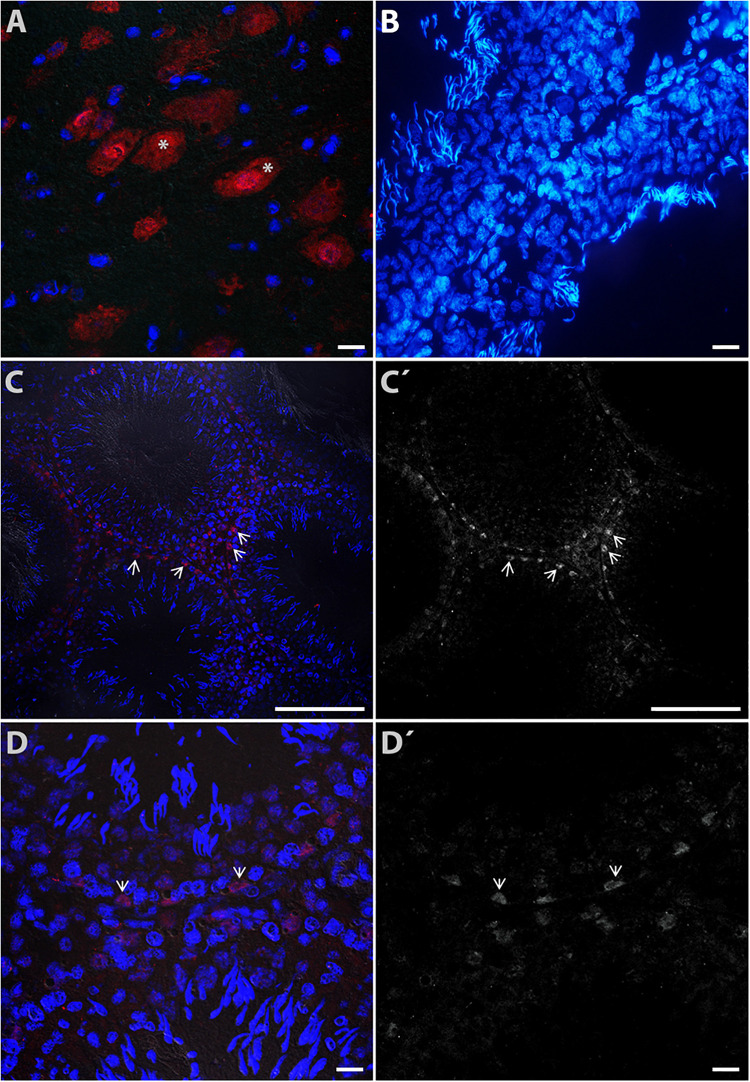
Digital photomicrograph illustrating the cytoplasmic distribution of VMAT1 immunoreactivity in rat testes. Neurons of the brain stem **(A)** were used a positive control (asterisks). The negative control is shown in **(B)** (counterstained with DAPI). Part **(C)** shows a panoramic view of detected immunoreactivity to VMAT1, mainly in Sertoli cells (arrowheads); **(C′)** shows the same view in grayscale (arrowheads indicate the same area). Part **(D)** shows a view of Sertoli cells intercalated between spermatogonia cells (arrow heads); **(D′)** is a duplicate image identifying the cellular phenotype.

For the vesicular transporter VMAT1, intense immunoreactivity was found in neurons of the brainstem used as a positive control (asterisks in Figure 5A). The negative control is shown in seminiferous tubules in Figure 5B (counterstained with DAPI). Figure 5C shows a panoramic view, with immunoreactivity to VMAT1 mainly in Sertoli cells (arrow heads); Figure 5C′ is a duplicate that shows the same view in grayscale (arrowheads indicate the same area). Figure 5D shows a close-up view of Sertoli cells intercalated between spermatogonia (arrowheads). Figure 5D′ shows a duplicate image in grayscale, used to identify the type of cells. Based on the captured images, we estimated intracellular expression using markers localized in different cell types (Table 1).

**TABLE 1:** Intercellular estimation of the different serotonin markers in the testes.

		Preleptotene					
Expression	Spermatogonial	Spermatocytes-like semestre	Spermatids round	Sperm	Sertoli cells	Myoid cells	Leydig cells
5-HT	+++	++	0	++	+	++	+++
TPH1	0	0	0	0	0	+	+++
MAO_A_	+++	++	+	+++	+	0	+
5-HT_T_	+	0	0	+++	+++	0	++
VMAT1	+	0	0	+	+++	0	0
5-HT_1B_	++	++	0	++	0	0	+++
5-HT_2A_	++	0	0	0	+	0	+
5-HT_3A_	+++	++	0	++	++	0	+

*+, some cells with expression; ++, increment in the number of cells observed; +++, mainly marked cells expression; 0, not observed.*

**FIGURE 6:**
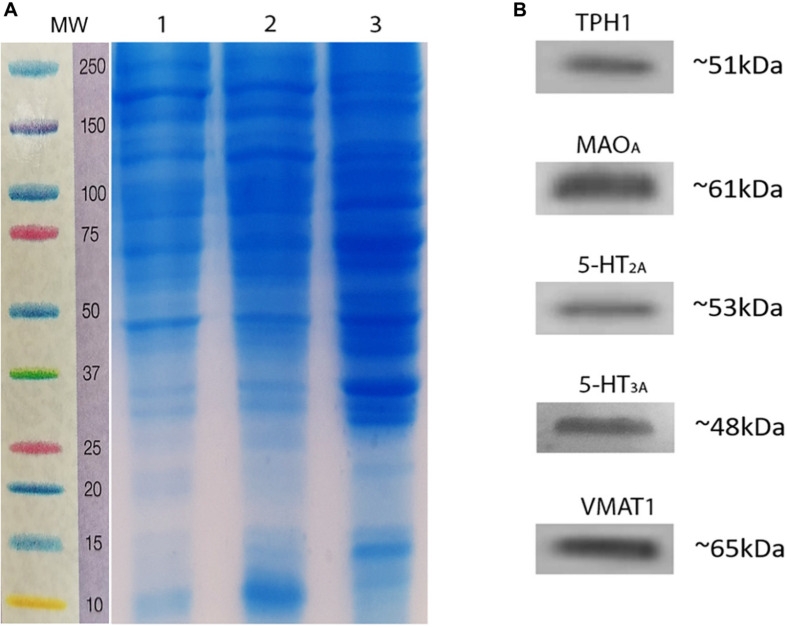
Immunodetection of serotoninergic system components in rat testes homogenates. **(A)** Marker ladder (MW, molecular weight) and representative control gel showing the presence of testes proteins. **(B)** Immunoblots using antibodies related to the serotoninergic system, showing a single band positive for TPH1, MAO_A_, 5-HT_2A_, 5-HT_3A_, and VMAT1.

To reconfirm the detection of 5-HT markers in homogenates of rat testes, we used western blot immunotransfer. Figure 6A shows a marker ladder and control gel with protein homogenates of adult rat testes of two animals (1, 2), and brain stem loaded in well 3 as a control for 5-HT system markers. The immunoblots in Figure 6B show the presence of serotoninergic components in rat testes. Positive bands appeared for both enzymes, TPH1 (∼51 kDa) and MAO_A_ (∼61 kDa), and for 5-HT_2A_ (∼53 kDa), 5-HT_3A_ (∼48 kDa), and V-MAT1 (∼65 kDa). Experiments were performed in triplicate (*n* = 3; see Supplementary Figures 3, 4).

By measuring the production of 5-hydroxytryptophan using HPLC, we confirmed the presence of TPH1 activity in rat testes: **∼0.6451 + 0.0613 nm/mg protein/h** (**∼0.5407 +0.1226**, relative to brain stem in rats). 5-HT concentration was also confirmed to be **∼206.814 + 0.4280 pg/mg tissue**. These results demonstrate the presence and activity of 5-HT in testes of 120-days-old rats (*n* = 7).

## Discussion

In the present work, we described the presence and localization of some components of the serotoninergic system and re-evaluated the presence of 5-HT in the testes of adult rats. 5-HT has previously only been detected in Leydig cells and the testicular capsule. Our results confirm that 5-HT is found in Leydig cells, as already described (Tinajero et al.,1993a,b; Frungieri et al., 1999), supporting the idea that this indolamine participates in the regulation of testosterone synthesis in these cells (Tinajero et al., 1993a). In addition, we found a regionalized signal of 5-HT inside the seminiferous tubules within peritubular myoid and spermatogonial cells. Blood capillaries, mast cells, and/or cytoplasmic prolongations of Leydig cells that reach the basal membrane could allow cells to capture 5-HT, as occurs in synaptic terminals and probably in caput epididymis (Jiménez-Trejo et al., 2007; Mohammad-Zadeh et al., 2008). Surprisingly, not all seminiferous tubules were positive for 5-HT; its distribution appeared to be regionalized in clusters of positive cells. Topographic and time-lapse microscopy studies should be carried out to analyze whether this distribution is dynamic or remains fixed.

As the concentration of 5-HT in tissues depends on its synthesis/catabolism ratio (Tyce, 1990), we evaluated the presence of both TPH1 and MAO_A_ enzymes in testes. We detected mRNA transcripts of Tph1 in whole testes, whereas TPH1 protein was mainly expressed in the interstitial zone, showing weak staining in cells close to the basal membrane. Jan et al. (2017) reconfirmed the expression of TPH1 in human spermatogonia by immunohistochemistry, suggesting that its RNA expression occurs in the spermatogonia.

Overall, this evidence supports the idea that 5-HT could be locally synthesized in Leydig cells (Tinajero et al., 1993a; Frungieri et al., 2002), where it could participate in the negative regulation of testosterone synthesis in an autocrine manner, as suggested by Tinajero et al. (1992). In addition, the putative presence of TPH1 and 5-HT that we found in the basal membrane of some seminiferous tubules suggests that 5-HT could participate in the regulation of spermatogenesis as well (see below).

MAO_A_ is a mitochondria-bound enzyme that catalyzes the degradation of monoamine neurotransmitters and dietary amines such as 5-HT by oxidative deamination. This enzyme catabolizes 5-HT into 5-hydroxyindole-3-acetaldehyde, which is further reduced by aldehyde reductase to 5-hydroxyindole-3-acetic acid. MAO_A_ may function in an autocrine manner to regulate proliferation and differentiation (i.e., prostatic epithelial cells; Gaur et al., 2019). We found its mRNA expressed in testes, with the protein located in the cytoplasm of Sertoli cells, spermatogonia, spermatocytes, and spermatid-like cells. The presence of this enzyme suggests that 5-HT could regulate fast processes in the seminiferous epithelium, which would require fast inactivation of this indolamine. Interestingly, in some tubules, we found MAO_A_ expressed along the flagellum of sperm localized in the seminiferous tubules, as has been reported in mature sperm in humans and horses (Jiménez-Trejo et al., 2012, 2018). Thus, MAO_A_ could be considered an early marker of maturation in sperm cells, an exciting possibility that should be investigated with further experiments.

A typical feature of 5-HT signaling is its remarkable complexity. This is mainly due to the high diversity of 5-HT receptors, which has been largely established over the past four decades (Gaspar et al., 2003; Göthert, 2013). We searched for 5-HT_1B_, 5-HT_2A_, and 5-HT_3A_ receptors in rat testes and found them all expressed in the seminiferous tubule, but with different patterns of distribution. 5-HT_1B_ was abundantly expressed in the basal region; 5-HT_2A_ was weakly stained in the basal region and more strongly in elongated processes that ran transversally to seminiferous tubules; and 5-HT_3A_ mRNA expression was found in whole testes, with the protein located in cell membranes of both spermatogonia and spermatocytes. 5-HT_1B_ and 5-HT_2A_ are metabotropic G protein-coupled receptors that can induce second messenger cascades (Gaspar et al., 2003; Adayev et al., 2005), whereas 5-HT_3_ is a ionotropic, ligand-gated ion channel that induces fast ionic responses (Na^+^, K^+^) (Gaspar et al., 2003; Mohammad-Zadeh et al., 2008). 5-HT receptors are known to induce proliferation, apoptosis, and differentiation in neurons and somatic cells (Gaspar et al., 2003; Adayev et al., 2005; Göthert, 2013). The presence of some subtypes in the seminiferous epithelium strengthens our idea of a controlled role of 5-HT in the spermatogenesis processes. Interestingly, *in vivo* and *in vitro* cell–cell interactions between Sertoli cells and germ cells regulate the secretion of germ cell development soluble factor(s), in this case, by meiotic pachytene spermatocytes that induce expression of 5-HT receptor mRNA (type 2). This occurs in rat Sertoli cells in culture and probably in testes, subject to regulation by endocrine and paracrine cues transmitted through Sertoli cells (Syed et al., 1999).

On the other hand, 5-HT_T_ and VMAT1 participate in 5-HT transport, although in different processes. 5-HT_T_ is an integral membrane solute carrier protein located mainly in the CNS but also in adrenal chromaffin cells, which recapture 5-HT for re-utilization and prevent permanent stimulation of signaling pathways through 5-HT receptors (Wang, 2004). VMAT1 is an integral membrane protein embedded in synaptic vesicles and large dense core vesicles (LDCVs) in neurons, and in large dense core granules (LDCGs) in neuroendocrine cells. It transfers monoamines between the cytosol and synaptic vesicles, LDCVs, or LDCGs, binding its substrates and internalizing them (Wimalasena, 2011). 5HT_T_ expression has been reported in the CNS (Daws et al., 2000), lung (Wang, 2004), caput epididymis (Jiménez-Trejo et al., 2007), gastrointestinal tract (Mazzawi and El-Salhy, 2016), pulmonary and peripheral vasculatures, and platelets (Mohammad-Zadeh et al., 2008). VMAT1 has been reported in adrenal chromaffin cells and enterochromaffin cells, which are responsible for storing 5-HT in the gastrointestinal tract (Hunyady et al., 2000). In the present work, we evaluated the presence and distribution of those transporters in rat testes. Surprisingly, we found both of them expressed in Sertoli cells; 5HT_T_ was also detected in Leydig cells and in the acrosomal region of sperms localized into the lumen, whereas VMAT1 was found principally in Sertoli cells. We found both MAO_A_ and 5HT_T_ expressed in sperm localized around the lumen of seminiferous tubules, suggesting that these cells have the machinery required to take up and degrade 5-HT.

This indolamine in sperm located in the lumen could induce an intracellular response, such as an increase in cytosolic Na^+^, which could interact with plasmalemma and/or mitochondrial Na^+^/Ca^2^+ exchangers, thus generating calcium signals during sperm capacitation prior to the fertilization process (Raiteri and Raiteri, 2015). The presence of VMAT1 in Sertoli cells agrees with our finding that 5-HT is found in such cells, where this transporter could promote its storage in vesicles. One possible explanation for this could be that VMAT1 facilitates the storage of other monoamines, such as dopamine (D2 receptors have been reported to be present in the seminiferous epithelium; Otth et al., 2007). The counts of cell expression by marker, generated through a table, allowed us to demonstrate variation in expression regardless of the serotonergic marker used, revealing in some cases a high cellular specificity.

Based on our results, we propose that a local serotoninergic system is present in rat testes, as has been previously described for caput epididymis and mature sperm, although with different functions (Jiménez-Trejo et al., 2007, 2012, 2018). The 5-HT, TPH1, and MAO_A_ enzymes, the 5-HT_1B_, 5-HT_2A_, and 5-HT_3A_ receptors, and the 5-HT_T_ and VMAT1 transporters were found to be expressed in different elements of the testes. They may contribute to the regulation of both steroidogenesis and spermatogenesis, which are key processes in testicular function. 5-HT is capable of activating several endocrine and paracrine factors, acting through non-cAMP-dependent pathways (Tinajero et al.,1993a,b), and it can also induce cytoskeleton rearrangements necessary for differentiation processes (Stephens and Prior, 1992). In this way, testicular cell populations, such as Sertoli cells, spermatogonia, and spermatids, are highly regulated in terms of their number and viability (Cooke et al., 1992). The distribution of the different elements of the serotoninergic system, as discussed above, suggests that 5-HT has an important role in this regulation. However, before concluding this and validating the earlier ideas, we must consider the specificity and sensitivity of the techniques that we have used. Although histochemistry may be considered an old method, this analysis has been used to identify not only 5-HT but also adrenaline, noradrenaline, and dopamine in testes, raising the possibility that other neurotransmitters could directly participate in the regulation of spermatogenesis in adult rats (Meizel, 2004; Hökfelt, 2010; Banerjee and Chaturvedi, 2019). The monoclonal antibodies against 5-HT that we used did not allow us to exclude the possibility of the presence of another indolamine, i.e., evidence of the local synthesis of melatonin in rat testes has been reported (Tijmes et al., 1996). However, the presence of mRNA, MAO_A_, and 5-HT receptors and transporters strengthens the idea that at least a fraction of 5-HT acts locally in both the interstitial space and seminiferous tubules.

Finally, we summarize our current knowledge about the multiple functions in which 5-HT is involved in the reproductive system. The description of a serotoninergic system in caput epididymis (Jiménez-Trejo et al., 2007) and mature sperm (Jiménez-Trejo et al., 2012), and now in testes, expands the possibilities for research on this important topic. It is clear that *in vivo* and *in situ* methodologies related to serotoninomics will help us to approach these important questions in a more functional way. It is worth remembering that the physiological role of 5-HT in the testes is still not fully understood. It is likely that 5-HT and its components represent an as-yet-unrecognized local inhibitory control mechanism related to blood vessels, steroidogenesis, and spermatogenesis in testicular function. However, further studies are required to assess the biological relevance of 5-HT in testes.

## Data Availability Statement

The datasets presented in this study can be found in online repositories. The names of the repository/repositories and accession number(s) can be found in the article/ Supplementary Material.

## Ethics Statement

The animal study was reviewed and approved by the Animal handling and experimentation followed the Guidelines for Care and Use of Laboratory Animals (CICUAL) and published by the National Institutes of Health.

## Author Contributions

FJ-T, RV-V, LH, and BR-R carried out the research. JR-C, CA-C, and MC-S analyzed the data. FJ-T, IC-M, and MC wrote the manuscript. All authors contributed to the article and approved the submitted version.

## Conflict of Interest

The authors declare that the research was conducted in the absence of any commercial or financial relationships that could be construed as a potential conflict of interest.

## Supplementary Material

Supplementary Figure 1 Complete gel for standardized RT-PCR; all RT-PCR amplicons are shown as well, as the molecular weight marker. Bs, brain stem; Te, testes.

Supplementary Figure 2 Gel containing the amplicons. The bands were taken from the gel showcased in the article to assemble the figure. It is practically the same result, except that the genes are in a different order compared with Supplementary Figure 1.

Supplementary Figure 3 Representative image of a complete electrophoresis gel of testicular protein homogenates. MW, molecular weight marker; T, testes; Bs, brain stem; Np, lane without protein.

Supplementary Figure 4 Representative image of the complete membrane, demonstrating immunoreactivity to the TPH enzyme. MW, molecular weight marker; Bs, brain stem; T, testes; T1/2, half the protein concentration; Np, lane without protein.

## Abbreviations

5-HT: serotonin
TPH: tryptophan hydroxylase
MAO_A_: monoamine oxidase A
PBS: phosphate buffer solution
CNS: central nervous system
5-HT_T_: serotonin transporter
VMAT1: vesicular transporter of monoamines
HPLC: high-performance liquid chromatography.
