# Supplementary figures and images for: Indolaminergic System in Adult Rat Testes: Evidence for a Local Serotonin System
Source: Front Neuroanat. 2021 Feb 19;14:570058. doi: 10.3389/fnana.2020.570058 (PMC7933592; doi:10.3389/fnana.2020.570058)

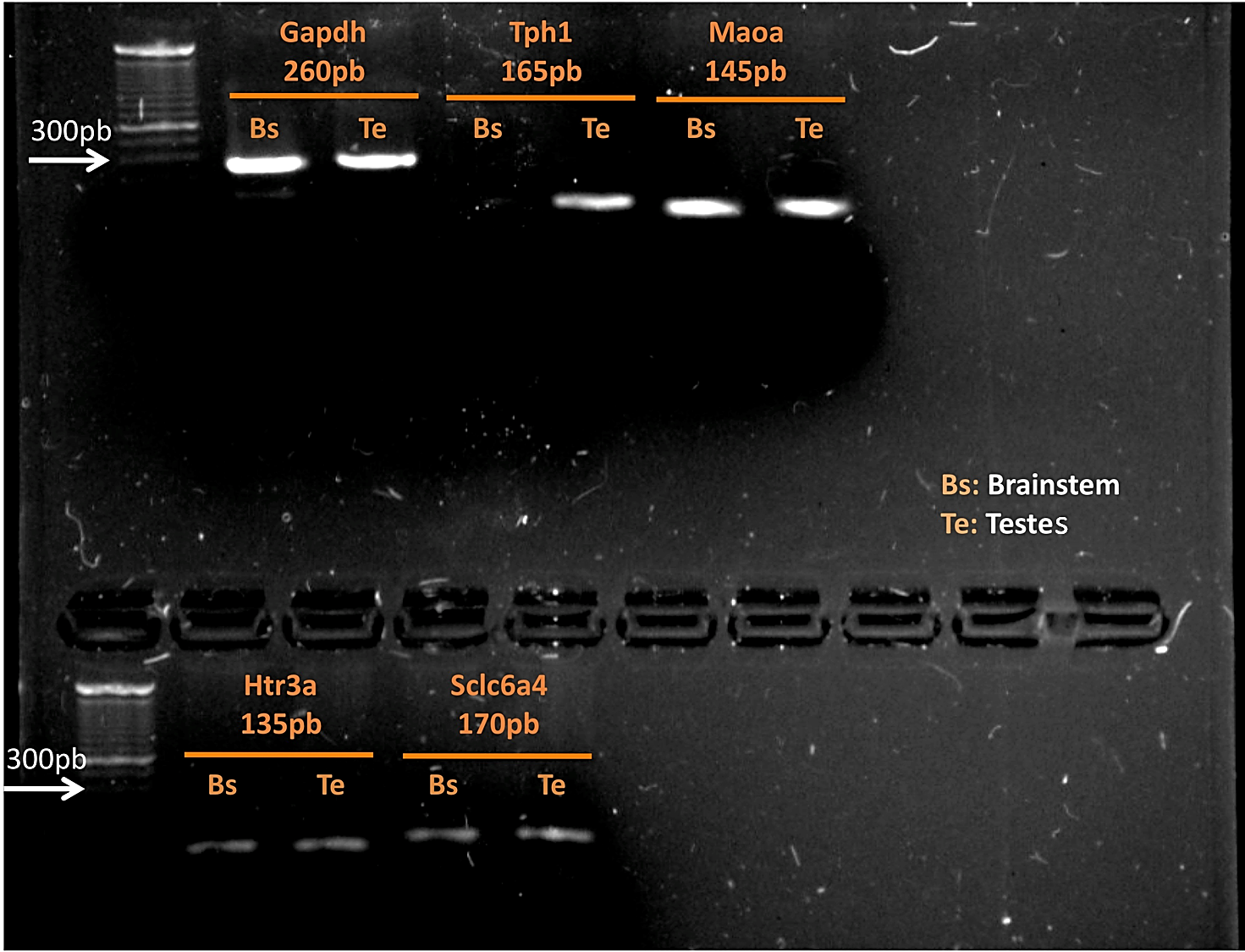

Supplement: Supplementary Figure 1 — Complete gel for standardized RT-PCR; all RT-PCR amplicons are shown as well, as the molecular weight marker. Bs, brain stem; Te, testes. [file Image_1.TIF]

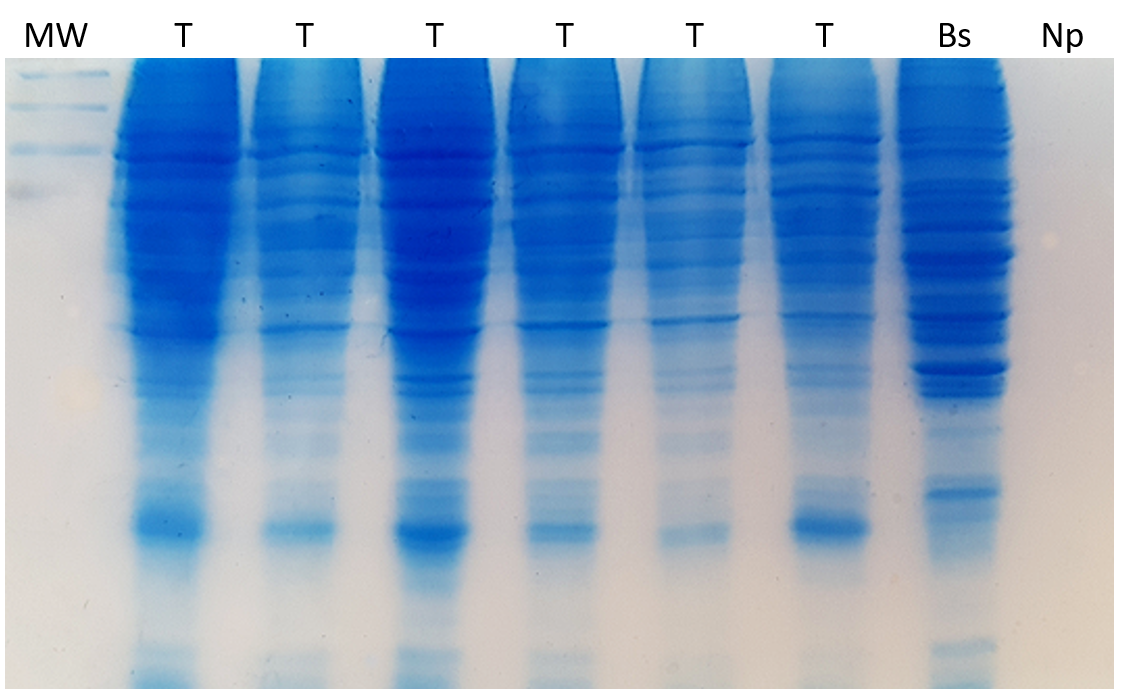

Supplement: Supplementary Figure 2 — Gel containing the amplicons. The bands were taken from the gel showcased in the article to assemble the figure. It is practically the same result, except that the genes are in a different order compared with Supplementary Figure 1. [file Image_2.TIF]

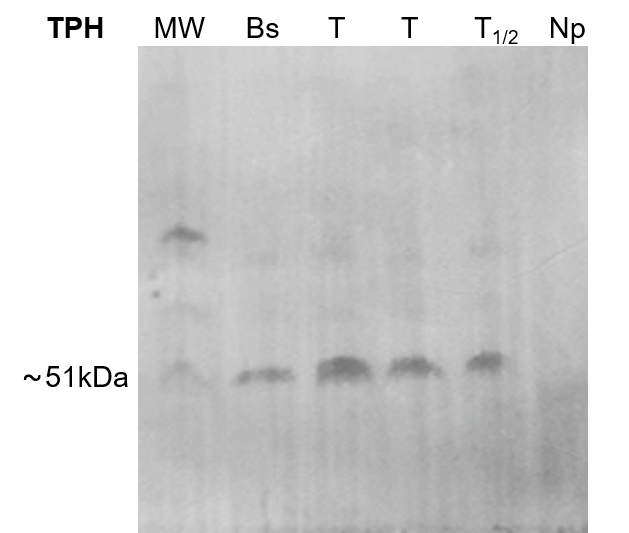

Supplement: Supplementary Figure 3 — Representative image of a complete electrophoresis gel of testicular protein homogenates. MW, molecular weight marker; T, testes; Bs, brain stem; Np, lane without protein. [file Image_3.TIF]

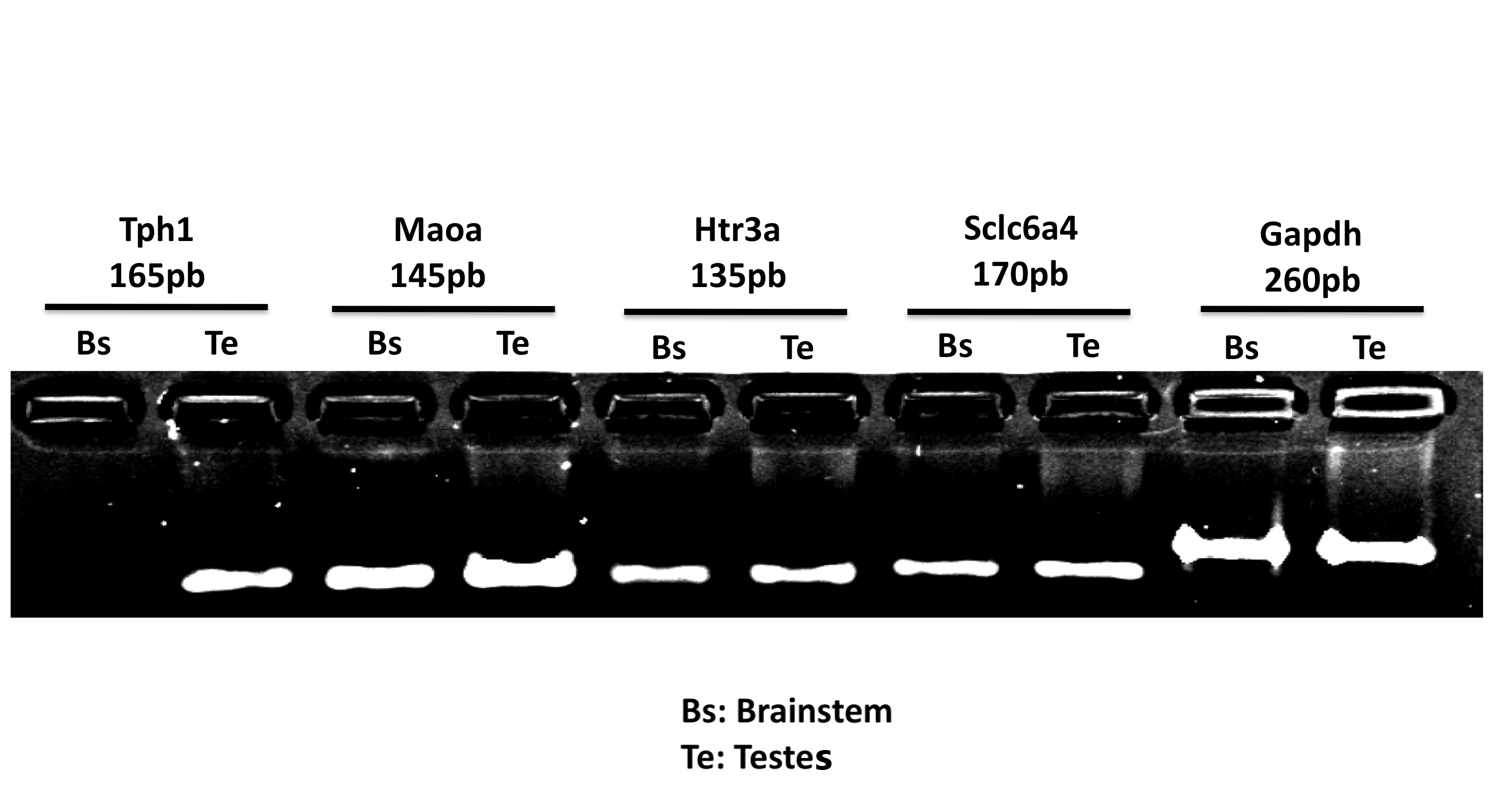

Supplement: Supplementary Figure 4 — Representative image of the complete membrane, demonstrating immunoreactivity to the TPH enzyme. MW, molecular weight marker; Bs, brain stem; T, testes; T1/2, half the protein concentration; Np, lane without protein. [file Image_4.TIF]
